# Intra-Arterial Drug and Light Delivery for Photodynamic Therapy Using Visudyne®: Implication for Atherosclerotic Plaque Treatment

**DOI:** 10.3389/fphys.2016.00400

**Published:** 2016-09-12

**Authors:** Manish Jain, Matthieu Zellweger, Aurélien Frobert, Jérémy Valentin, Hubert van den Bergh, Georges Wagnières, Stéphane Cook, Marie-Noelle Giraud

**Affiliations:** ^1^Cardiology, Department of Medicine, University and Hospital of FribourgFribourg, Switzerland; ^2^Medical Photonics Group, LCOM-ISIC, Swiss Federal Institute of Technology (EPFL)Lausanne, Switzerland

**Keywords:** photodynamic therapy, visudyne®, verteporfin, atherosclerosis, macrophage, apoptosis

## Abstract

Photodynamic therapy (PDT), which is based on the activation of photosensitizers with light, can be used to reduce plaque burden. We hypothesized that intra-arterial photosensitizer administration and photo-activation will lead to high and rapid accumulation within the plaque with reduced systemic adverse effects. Thus, this “intra-arterial” PDT would be expected to have less side effects and due to the short time involved would be compatible with percutaneous coronary interventions.

**Aim:** We characterized the dose-dependent uptake and efficacy of intra-arterial PDT using Liposomal Verteporfin (Visudyne®), efficient for cancer-PDT but not tested before for PDT of atherosclerosis.

**Methods and Results:** Visudyne® (100, 200, and 500 ng/ml) was perfused for 5–30 min in atherosclerotic aorta isolated from ApoE^−/−^ mice. The fluorescence Intensity (FI) after 15 min of Visudyne® perfusion increased with doses of 100 (FI-5.5 ± 1.8), 200 (FI-31.9 ± 1.9) or 500 ng/ml (FI-42.9 ± 1.2). Visudyne® (500 ng/ml) uptake also increased with the administration time from 5 min (FI-9.8 ± 2.5) to 10 min (FI-23.3 ± 3.0) and 15 min (FI-42.9 ± 3.4) before reaching saturation at 30 min (FI-39.3 ± 2.4) contact. Intra-arterial PDT (Fluence: 100 and 200 J/cm^2^, irradiance-334 mW/cm^2^) was applied immediately after Visudyne® perfusion (500 ng/ml for 15 min) using a cylindrical light diffuser coupled to a diode laser (690 nm). PDT led to an increase of ROS (Dihydroethidium; FI-6.9 ± 1.8, 25.3 ± 5.5, 43.4 ± 13.9) and apoptotic cells (TUNEL; 2.5 ± 1.6, 41.3 ± 15.3, 58.9 ± 6%), mainly plaque macrophages (immunostaining; 0.3 ± 0.2, 37.6 ± 6.4, 45.3 ± 5.4%) respectively without laser irradiation, or at 100 and 200 J/cm^2^. Limited apoptosis was observed in the medial wall (0.5 ± 0.2, 8.5 ± 4.7, 15.3 ± 12.7%). Finally, Visudyne®-PDT was found to be associated with reduced vessel functionality (Myogram).

**Conclusion:** We demonstrated that sufficient accumulation of Visudyne® within plaque could be achieved in short-time and therefore validated the feasibility of local intravascular administration of photosensitizer. Intra-arterial Visudyne®-PDT preferentially affected plaque macrophages and may therefore alter the dynamic progression of plaque development.

## Introduction

Acute coronary syndrome (ACS) is a life-threatening condition resulting from rupture of vulnerable atherosclerotic plaque (Gotlieb, 2005). The optimal medical therapy currently available with statins prevents only 20–30% of cardiovascular events (Kapur and Musunuru, 2008). Dual anti platelet therapy (DAPT) with aspirin plus clopidogrel, prasugrel or ticagrelor, for up to 12 months after ACS has been shown to improve outcomes after the cardiovascular event (Yusuf et al., 2001; Davis et al., 2015). However, DAPT is often associated with increased risk of bleeding (Clappers et al., 2007; Fanari et al., 2016). Percutaneous coronary intervention (PCI) with balloon angioplasty, often followed by stent implantation is the most commonly performed procedure for the reopening of an occluded artery. Besides restenosis and stent related problems (Kuchulakanti et al., 2006; Gogas et al., 2014), this procedure is mainly used on highly stenosed plaques. Less stenosed plaques, diffused over long segments of artery, are often left untreated. These untreated plaques continue to evolve and may eventually rupture (Skowasch et al., 2005; Moulias and Alexopoulos, 2011). Further interventions, either standalone or in conjunction with existing procedures, are emerging for management of atherosclerosis (Kalanuria et al., 2012; Riccioni and Sblendorio, 2012). Among them, photodynamic therapy (PDT) has been shown to prevent atherosclerotic plaque progression (Usui et al., 1999; Peng et al., 2011). PDT generally involves either topical or, mostly, systemic administration of a photosensitizer which is allowed to circulate for a specific period of time to accumulation, in our case, in atherosclerotic lesions. This time period is often referred to as drug-light interval (DLI). Activation of the photosensitizer with visible light of a specific wavelength generates cytotoxic reactive oxygen species (ROS; Zhu et al., 2015). These ROS oxidize essential tissue components and can eventually induce cell death in tissue. Application of PDT for atherosclerotic plaque treatment has faced some drawbacks due to the non-specific accumulation of the photosensitizer in the skin, leading to cutaneous photosensitivity (Wooten et al., 1988; Dougherty et al., 1990; Wagnieres et al., 1998). Another major hurdle for the clinical use of PDT for cancerous tissue (Braichotte D. et al., 1995; Braichotte D. R. et al., 1995; Zellweger et al., 2000) or particularly for the management of atherosclerosis is long DLI (from 3 to 24 h) after systemic injection with most of the tested PSs (Kereiakes et al., 2003; Tawakol et al., 2006). This puts PDT out of the scope for cases where it is used alongside the treatment of acute myocardial infarction. A short DLI is therefore critically important for its applicability to treat those cases.

Besides DLI, accurate and efficient delivery of light is also important for the activation of photosensitizers. Previous reports indicated that external illumination of the artery using Talaporfin sodium as a photosensitizer resulted into photo toxicity of the irradiated site, however the contralateral walls show limited photo toxicity (Wakamatsu et al., 2005). Intra vascular irradiation circumvents this shortcoming. Intra-arterial photo activation enables circumferential, controlled, and homogeneous illumination of the atherosclerotic artery. In addition, illumination length with our present setup can vary from 1 to 7 cm and even longer light/drug applications are possible. This permits to treat much longer segments of the artery as compared to stents. We hypothesized that local delivery of photosensitizer using an intravascular catheter followed by intravascular illumination may be performed during or just after a PCI procedure and stent implantation. Thus, this “intra-arterial PDT” would be expected to have fewer side effects and due to the short time involved would be compatible with PCI procedures. Combining PCI with PDT may reduce restenosis and may permit the preventive treatment of non-stented vulnerable plaque in order to reduce further coronary events. Furthermore, as the main parameter of PDT is the selective drug uptake in sufficient quantity in given location of the lesion, this can likely be improved by local drug delivery.

Verteporfin, also known as benzoporphyrin derivative monoacid ring A is a second generation photosensitizer. Visudyne® (Novartis) is a liposomal formulation of Verteporfin with a short DLI and is clinically utilized for the treatment of age related macular degeneration and polypoidal choroidal vasculopathy. Previous reports highlight that Verteporfin can accumulate in plaque (Hsiang et al., 1993; Allison et al., 1997). However, studies demonstrating the therapeutic potential of Verteporfin upon photo activation for the treatment of atherosclerotic plaque are still lacking. The present study aims to examine the effect of Visudyne® mediated PDT on atherosclerotic plaque as well as optimization of the local photosensitizer and light delivery within atherosclerotic plaque to reduce the side effects of treatment.

## Materials and methods

### Chemicals

Dihydroethidium (DHE), Terminal Transferase from Calf Thymus (TdT), Digoxigenin-11-UTP, Anti-Digoxigenin-Rhodamine Fab fragments, phenylephrine, Acetylcholine, Haematoxylin, Eosin, Oil Red O, and other fine chemicals were purchased from Sigma (Buchs, Switzerland). Primary antibodies including F4/80, α-smooth muscle actin, Von Willebrand factor, appropriate secondary antibodies conjugated to Alexa Fluor 488/546 and Hoechst were obtained from Abcam (Cambridge, UK).

### Photosensitizer

Visudyne® (PubChem CID:5362420) is commercially available (Novartis) and 1 g Visudyne® powder consisted of 15 mg Verteporfin as active photosensitizer. Visudyne® was dissolved in 0.9% NaCl and 5% glucose (B. Braun Medical AG, Sempach) at a concentration of 100, 200, or 500 ng/ml that actually contained 1.5, 3, or 7.5 ng/ml of Verteporfin. Solutions of the photosensitizers were prepared fresh and used within 1 h.

### Animal experiments

Seven weeks old male ApoE^−/−^ mice with the C57BL/6 genetic background weighing 21 ± 2 g were obtained from Charles River laboratories, France. The animals were housed in individually ventilated cages in the animal center facility at University of Fribourg (Switzerland). All animals received humane care and in compliance with the European Convention on Animal Care in accordance with the Swiss Animal Protection Law after obtained permission of the State Veterinary Office, Fribourg and approved by the Swiss Federal Veterinary Office, Switzerland (FR 2013/35). Animals were maintained on chow diet and water *ad libitum* in a controlled environment at a temperature of 20°C with 40–50% humidity, 12-h/12-h light/dark cycle. After 1 week of adaptation, ApoE^−/−^ mice were fed for 16 to 20 weeks with a lipid-rich diet (0.2% cholesterol and 21% butter, Western U8958 version 35, SAFE, France).

### Atherosclerotic plaque characterization

Animals were euthanized; thoracic aorta was dissected and embedded in Tissue-Tek O.C.T. compound according to standard procedure. Five micrometers thick serial sections were cut and stained with Haematoxylin-Eosin (HE) to characterize the general architecture of the plaques. Intima thickness, media thickness, necrotic core area and plaque area was determined on HE stained sections (Khanna et al., 2013; Jain et al., 2015). The lesion was traced manually and measured using ImageJ software. Plaque area was defined as the area between innermost elastin lamina and lumen of the artery. Necrotic core area was defined as acellular areas in the intima and is represented as % necrotic core area/plaque area (Thorp et al., 2009). Plaque lipid was determined by Oil red O staining and is represented as % Oil red O positive area/plaque area (Khanna et al., 2013). Morphometric analysis was performed using bright field microscopy by a Nikon Ni-U microscope (Nikon, Tokyo, Japan) utilizing the 4x and 20x objectives. Plaque smooth muscle cell and macrophage content was determined by immunohistochemical analysis. Briefly, aortic sections were permeabilized using 0.2% triton and incubated for 1 h with α-SM actin (Abcam, 1:200) or F4/80 antibody (Abcam, 1:200) to detect smooth muscle cells (SMC) and macrophages respectively. Staining was quantified using the ImageJ software as previously described (Jensen, 2013).

### Intra-arterial drug delivery

The mice were anesthetized and sacrificed using Isoflurane in accordance with an approved protocol. Thoracic aorta (1.5 cm length) was excised, cleaned and mounted on a cannula made from blunted 25-gauge hypodermic needles and fixed within a stainless steel support. The whole assembly was then placed in the vessel chamber containing Krebs bicarbonate solution (composition in mM: NaCl 118; KCl 5; CaCl_2_ 2.5; MgSO_4_ 1.2; KH_2_PO_4_ 1.2; NaHCO_3_ 25; glucose 11; EDTA 0.03; pH 7.4) maintained at 37°C. The vessel holding cannula was connected to a syringe pump (World Precision Instruments, Sarasota, FL) and arteries were perfused in the dark with Visudyne® (100–500 ng/ml) or vehicle (0.9% NaCl and 5% glucose) for 5–30 min at a flow rate of 1.2 ml/min (Hayase et al., 2001). The medium was constantly bubbled with 5% CO_2_ and 95% O_2_ (Hitchcock et al., 2010; Prasad et al., 2014). After perfusion, aortic segments were immediately flushed with vehicle and were embedded in O.C.T. compound using liquid nitrogen

### Visudyne® uptake

The localization of Visudyne® within the artery was assessed on histological sections (5 μm) using a Leica TCS SP5 DMI6000 confocal microscope (Leica Microsystems) equipped with Leica plan apo 20 × (numerical aperture 0.7) dry objective. The sections were illuminated with 405 nm Diode laser. Images were collected in the fluorescence emission range of 650–750 nm. Elastin auto fluorescence was co-detected upon excitation with 488-nm laser light. Pictures were taken by using Leica Application Suite Advanced Fluorescence (LASAF) software (Leica Microsystems). Mean fluorescence intensity was quantified and normalized with respect to the area of tissue using the ImageJ software as previously described (Jensen, 2013).

### Light source and delivery

Intra-arterial PDT was performed immediately after Visudyne® (500 ng/ml) perfusion using a cylindrical light diffuser that is an optical fiber based catheter (RD20, Medlight SA, Switzerland). The proximal end of the diffuser is coupled to a 1W Diode-pumped solid-state laser (Frankfurt Laser Company, Germany) emitting at 690 nm. The distal extremity of diffuser is a 20 mm long illumination tip. Laser powers emitted by the cylindrical light diffusers were calibrated using an integration sphere, by comparison with a frontal light diffuser (FD-1, Medlight SA, Switzerland) delivering 150 mW measured with a power-meter (Spectra-Physics Newport; model 407A; Borle et al., 2003). The illumination tip of the light diffuser was inserted into the lumen of the artery via the cannula through which Visudyne® was perfused. Total light doses of 100 J/cm^2^ or 200 J/cm^2^ were applied at an irradiance of 334 mW/cm^2^. After PDT, the aorta was removed from the cannula and divided into two parts. The proximal part was immediately snap-frozen and used for ROS detection. The distal part was kept in Dulbecco's Modified Eagle Medium supplemented with 10% FBS and antibiotics (100 U/ml penicillin and 100 μg/ml streptomycin) and incubated at 37°C for 24 h to assess the influence of PDT on apoptosis, a late-stage event.

**Figure 1:**
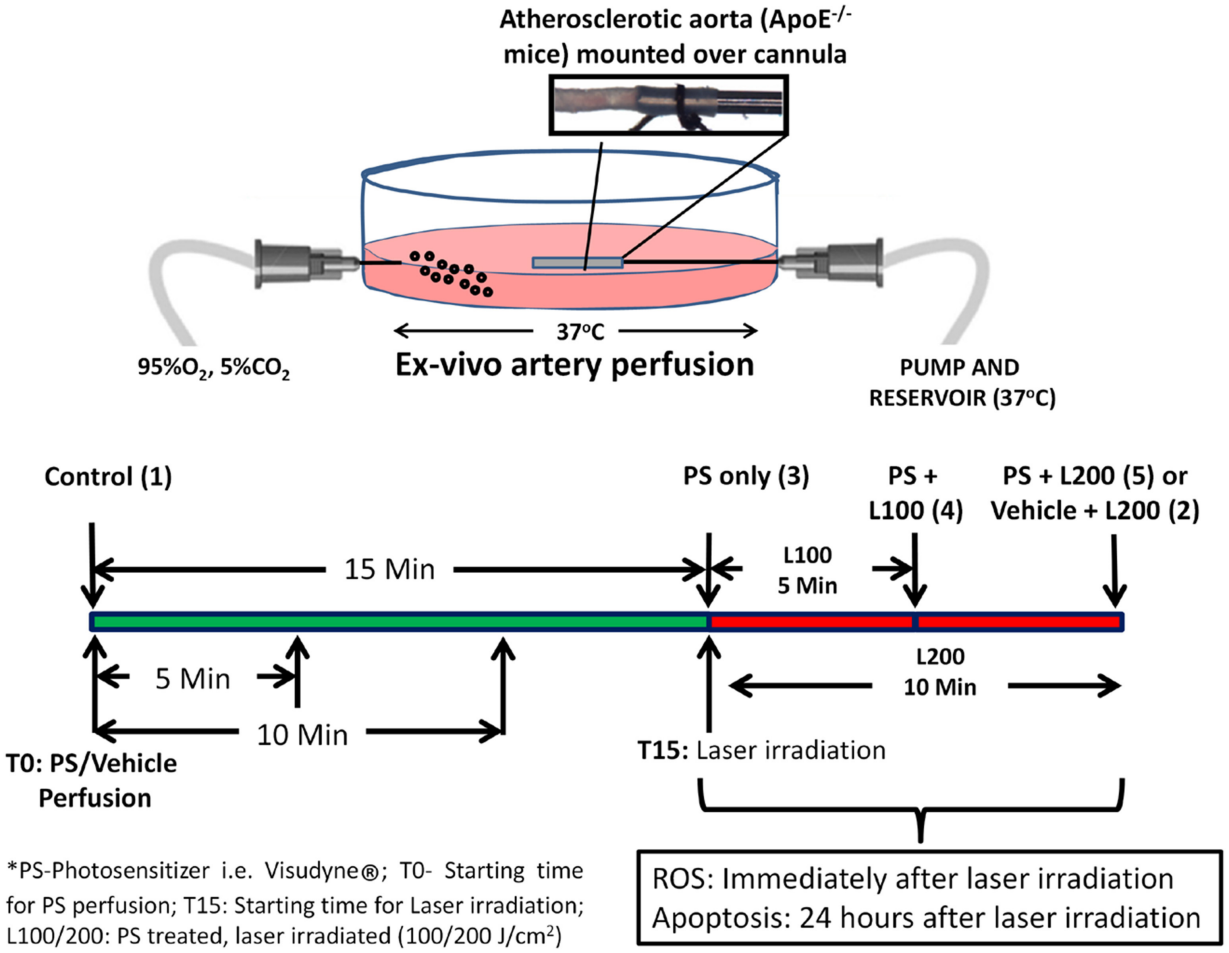
**Schematic representation of the *ex-vivo* artery perfusion system where atherosclerotic aorta, isolated from ApoE^−/−^ mice, was mounted over a cannula and perfused with vehicle or Visudyne^®^ for 5–30 min**. Laser irradiation at 100 or 200 J/cm^2^ was performed immediately after perfusion. ROS level and apoptosis was monitored immediately or after 24 h of laser irradiation respectively.

To characterize the dose and time related uptake of Visudyne® as well as to assess the influence of Visudyne®-PDT, laser irradiations only or combination of both on atherosclerotic plaque, animals were divided into (1) Vehicle (5% glucose in normal saline) treated control, (2) Vehicle treated, laser irradiated (200 J/cm^2^) group, (3) Non-irradiated Visudyne® treated groups, (4) Visudyne® treated, laser (100 J/cm^2^) irradiated group (5) Visudyne® treated, laser (200 J/cm^2^) irradiated group (Figure 1).

### Detection of ROS

Aortic superoxide content was determined by Dihydroethidium (DHE) staining. In the presence of superoxide, DHE is oxidized to ethidium that intercalates with DNA and becomes fluorescent. Briefly, aorta sections were rinsed with PBS and incubated with DHE (10 μM) in a dark humidified chamber for 10 min at 37°C. The sections were then washed with PBS and counterstained with Hoechst (5 μg/mL; He et al., 2016). Fluorescent images were acquired with same exposure time for all sections from different groups on a Nikon Ni-U microscope (Nikon, Tokyo, Japan). DHE fluorescence density was measured using ImageJ software after subtracting auto-fluorescence from elastic lamellae (Raaz et al., 2015).

### Detection of apoptotic cells by tunel staining

Apoptotic cells within the aortic segments were detected by terminal deoxynucleotidyl transferase-mediated dUTP nick-end labeling (TUNEL) 24 h after PDT (Gad et al., 2001). Briefly, aortic sections were permeabilized with 0.2% Triton and subsequently incubated for 30 min at 37°C in a humidified chamber in the TUNEL reaction mixture containing Digoxigenin-11-UTP. Incorporated digoxigenin was detected using rhodamine-conjugated anti-digoxigenin antibody. Apoptotic cells were detected and quantified using ImageJ software as described previously (Veurink et al., 2003). Percentage of apoptotic cells/total cells was determined in the entire section, and either only in plaque or only in media beneath plaque. For identification of apoptotic macrophages, double-immunostaining was performed as described previously (Ray et al., 2000). In brief, after TUNEL staining, sections were rinsed with PBS and incubated with primary antibody for F4/80 (1:100) at room temperature for 60 min. The sections were washed and labeled with appropriate secondary antibody conjugated with Alexa fluor 488 for 30 min followed by staining of nuclei with Hoechst (5 μg/mL) for 10 min. The sections were examined under a fluorescence microscope and the percentage of apoptotic macrophages of the total number of cells was determined. Two fields were selected to cover almost entire tissue section.

### Assessment of vascular function

Vascular reactivity was assessed after 24 h on arterial segments treated in a similar way as described above. After 24 h of treatment, aortic segments were cut into rings (2 mm in length) and mounted between two L shaped hooks in a Multi-Myograph System (Model 720 MO, Danish Myo Technology A/S, Denmark). Myograph chambers were filled with Krebs bicarbonate buffer and were bubbled with 95% O_2_ and 5% CO_2_ at 37°C. Changes in isometric tension were recorded with Lab Chart Pro v8.0.5 software (AD instruments, UK). The rings were progressively stretched to a passive tension of 5 mN and were allowed to equilibrate for 60 min during which the Krebs buffer was changed every 15 min. The resulting tension after equilibration period represented the base line value. In order to assess the maximum tissue contractility and viability, the rings were maximally contracted with 80 mM KCl Krebs solution and change in tension (ΔmN) was recorded. After the assessment of maximum tissue contractility, rings were washed and rested for 15–20 min so that tension returns to the baseline. Cumulative dose response curve to phenylephrine (PE; 1 nm–100 μM) was then taken (Khanna et al., 2011; Jain et al., 2012). Vasoconstriction response to PE was expressed as the increase in force (ΔmN) from base line upon cumulative addition of different doses of PE. To assess endothelium dependent relaxation, aortic rings were washed and subsequently pre-constricted with a submaximal concentration of PE (inducing 60–80% of maximal vasoconstriction). Rings were then exposed to cumulative doses of Acetylcholine (Ach; 3 nM–300 μM; Jain et al., 2016). Relaxation response to Ach was expressed as the net decrease in force (ΔmN) generated by submaximal PE. Vasoconstriction and vasodilation responses were normalized with respect to per mm length of aortic rings. Since in the present study, smooth muscle contraction was substantially reduced, we also calculated percentage relaxation (decrease in submaximal PE induced contraction upon addition of Ach/Submaximal PE induced contraction × 100) in order to normalize the reduced contractility.

### Statistical analysis

All experiments were performed at least three times. The results are presented and expressed as the Mean ± Standard deviation. The significance of difference among groups was analyzed by one way analysis of variance (ANOVA) followed by Bonferroni *post-hoc* test using GraphPad Prism version 5.0 (GraphPad Software, La Jolla, CA). *p* < 0.05 were considered statistically significant.

## Results

### Analysis of plaque composition

**Figure 2:**
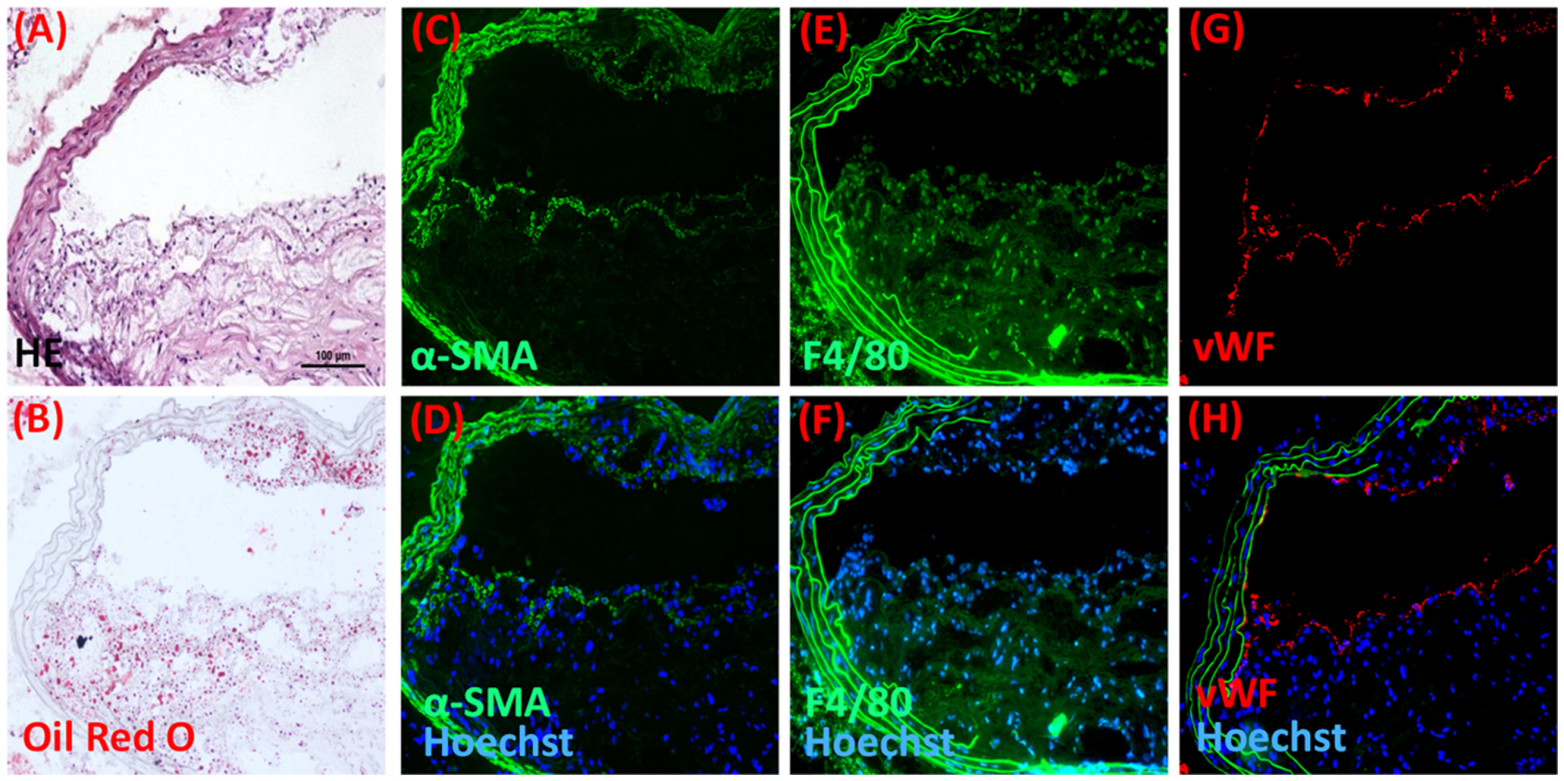
**Plaque composition in ApoE^−/−^ after 16-20 weeks of lipid-rich diet feeding**. Representative microscopic images stained with **(A)** hematoxylin-eosin and **(B)** Oil red O demonstarting general architecture and lipid content of the plaque respectively. Plaques were analyzed by immunohistochemistry for the presence of **(C,D)** α-SM-actin, **(E,F)** Macrophage (F4/80), and **(G,H)** endotheium (vWF), where **(D,F,H)** are respective images merged with green elastin auto fluorescence and blue fluorescence of Hoechst stained nuclei. Scale bar in *A* = 100 μm and applies to **(A–H)** (*n* = 7).

Localization of the photosensitizer within the atherosclerotic plaque could vary significantly depending on multiple parameters including the type of lesion, its location and stage of development. We therefore characterized the plaque composition within the thoracic aorta of ApoE^−/−^ mice. Gross examination of the atherosclerotic plaques used in the present study revealed high plaque lipid content, large necrotic cores, few calcium deposits, and medial destruction (Figure 2, Table 1). Immunohistochemistry data demonstrated that the lesions mainly consisted of macrophage with well-formed but thin fibrous cap containing SMC and the lesions were lined with endothelial cells (Figure 2, Table 1).

**Table 1:** **Plaque composition from thoracic aorta sections of ApoE^−/−^ mice fed with a lipid-rich diet for 16–20 weeks**.

**Intima thickness (μm)**	**Media thickness (μm)**	**Intima/media thickness ratio**	**% Necrotic core area**	**Plaque area (μm^2^)**	**% Oil red O positive area**	**% Plaque SMC**	**% Macrophage**
174.4 ± 51.7	47.2 ± 6.8	3.9 ± 1.2	17.8 ± 3.2	11168 ± 2705	23.0 ± 5.8	21.5 ± 6.2	63.4 ± 11.1

Results are mean ± SD from (n = 7).

### Dose dependent accumulation of Visudyne®

**Figure 3:**
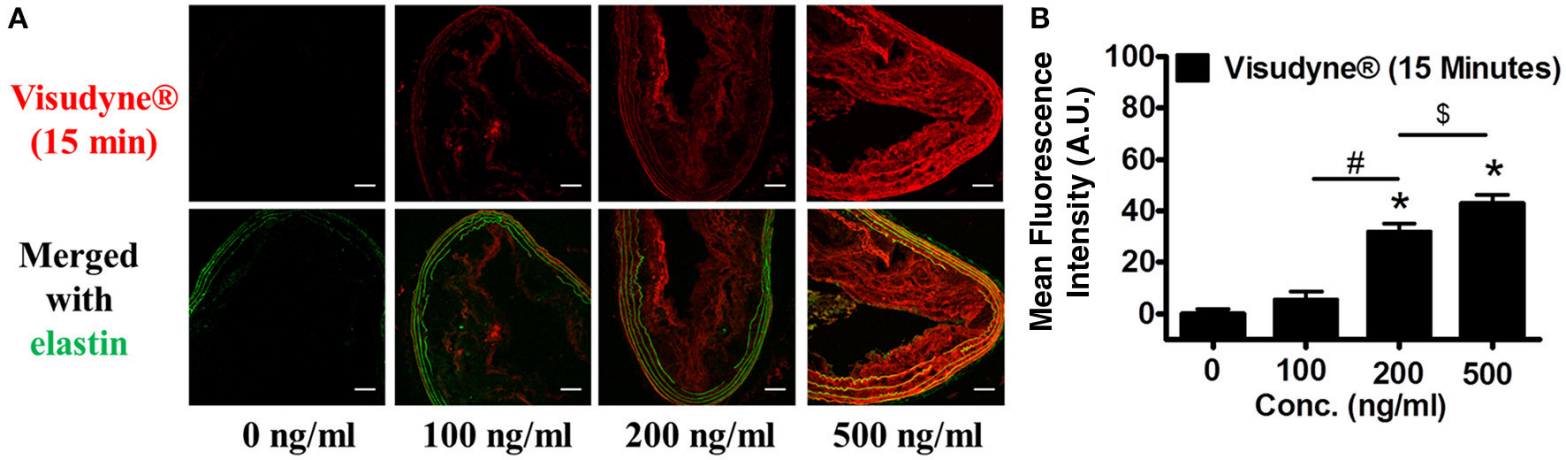
**Dose dependent Visudyne^®^ uptake**. Excised aorta from ApoE^−/−^ mice were perfused for 15 min with 0 (*n* = 6), 100 (*n* = 3), 200 (*n* = 3), or 500 ng/ml (*n* = 8) of Visudyne®. **(A)** Arterial sections were excited with 405 nm light and fluorescence emission was measured between 650 and 750 nm. Upper panel illustrates characteristic fluorescence of Visudyne® in red and lower panel is merged image with auto-fluorescence of elastin. Scale bar: 50 μm. **(B)** Bar graph representing the Mean fluorescence intensity in each group. *P < 0.001 vs. 100 ng/ml, ^#^*p* < 0.001 100 ng/ml vs. 200 ng/ml, ^$^*p* < 0.001 200 ng/ml vs. 500 ng/ml.

To determine the optimal dose of photosensitizer, three concentrations of Visudyne® i.e., 100, 200, and 500 ng/ml (1.5, 3.0, and 7.5 ng/ml Verteporfin as active photosensitizer) were perfused in the isolated aorta for 15 min. Fluorescence imaging of the artery sections demonstrated characteristic fluorescence of Visudyne® (Figure 3A). Exposure of the atherosclerotic aorta to increasing concentrations of Visudyne® demonstrated a dose dependent increase in fluorescence intensity (Figure 3B). Weak fluorescence observed at 100 ng/ml (FI: 5.5 ± 3.2) was enhanced by 4.8-fold at 200 ng/ml (F: 31.9 ± 3.2) or 6.8-fold at 500 ng/ml (FI: 42.9 ± 3.4). Visudyne® uptake at 500 ng/ml concentration was significantly higher than 200 ng/ml concentration and was therefore used for further studies.

### Time-dependent Visudyne® accumulation

**Figure 4:**
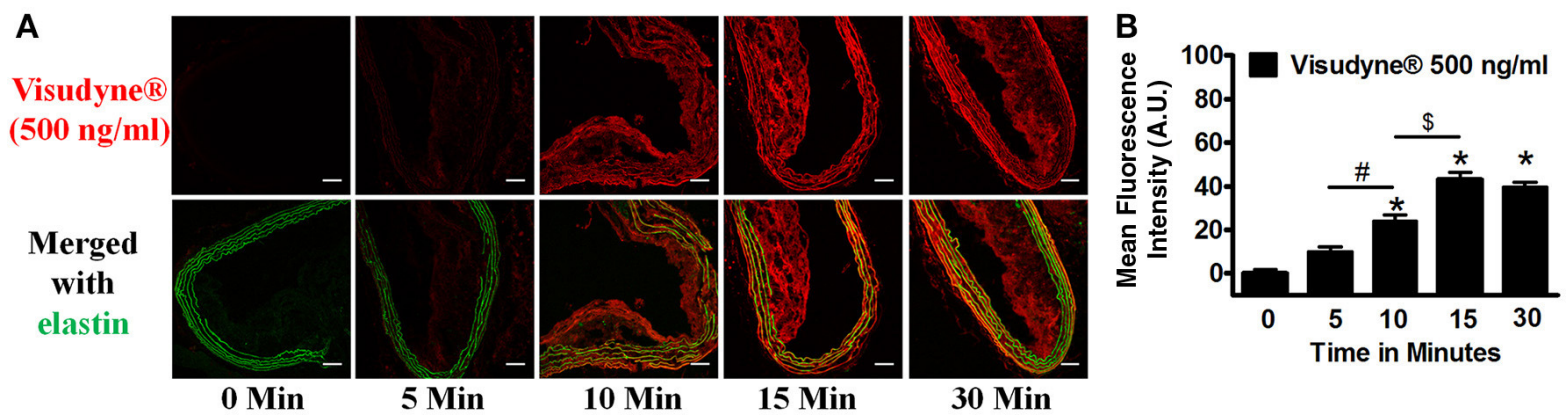
**Time dependent Visudyne^®^ uptake**. Aorta isolated from ApoE^−/−^ mice were treated with Visudyne® (500 ng/ml) at different time intervals. **(A)** Representative sections demonstrating Visudyne® accumulation at 5 (*n* = 4), 10 (*n* = 4), 15 (*n* = 8) and 30 min (*n* = 4). Scale bar: 50 μm. **(B)** Bar graph representing the mean fluorescence intensity after 5–30 min of treatment. *P < 0.001 vs. 5 min, ^#^*p* < 0.001 5 vs. 10 min, ^$^*p* < 0.001 10 min vs. 15 min.

Perfusion with Visudyne® (500 ng/ml) demonstrated a time-dependent increase in fluorescence (Figure 4A). Quantification of the fluorescence intensity revealed Visudyne® accumulation as early as after 5 min of treatment (FI: 9.8 ± 2.5). This accumulation increased at 10 min (FI: 23.7 ± 3.0). The maximal fluorescence was observed after 15 min of treatment (FI: 42.9 ± 3.4), while 30 min exposure showed no further increase in fluorescence (FI: 39.3 ± 2.4; Figure 4B). Since FI after 15 min was comparable to 30 min treatment, we have therefore chosen the 15 min time point as optimal treatment time for the light activation.

### Visudyne®-PDT stimulated ROS generation

**Figure 5:**
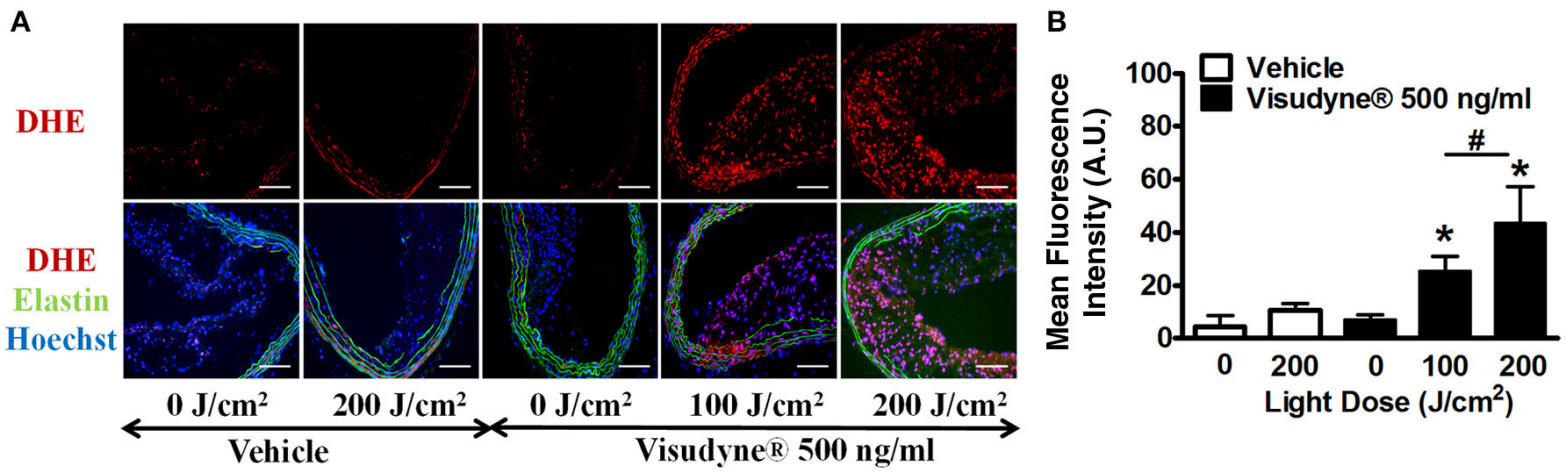
**ROS production by Visudyne^®^ mediated PDT. (A)** Fluorescent photomicrograph of Dihydroethidium staining in aortic sections from vehicle (*n* = 6), laser only (*n* = 4), Visudyne® only (*n* = 4), Visudyne® treated, laser (100 J/cm^2^; *n* = 6) or Visudyne® treated, laser (200 J/cm^2^; *n* = 6) treated groups. Upper panel shows ROS signals as red fluorescence and lower panel represents the merged image with green elastin auto fluorescence together with blue fluorescence of the Hoechst stained nuclei. Scale bar: 100 μm. **(B)** Bar diagram representing DHE fluorescence intensity/μm^2^. *P < 0.05 vs. 0 J/cm^2^, ^#^*p* < 0.01 100 vs. 200 J/cm^2^.

ROS levels were monitored using DHE staining (Figure 5A). DHE fluorescence intensity in vehicle treated, laser irradiated (200 J/cm^2^; FI: 10.6 ± 2.6) was slightly increased as compared to only vehicle treated control (FI: 4.5 ± 4.0). ROS level in Visudyne® treated groups was not affected when the irradiation dose was zero (FI: 6.9 ± 1.8). A 4.5-fold increase in ROS levels was observed in Visudyne® photo irradiated arteries at 100 J/cm^2^ (FI: 25.3 ± 5.5) as compared to vehicle treated control (Figure 5B). ROS levels further increased by 8.4-fold upon increasing the light irradiation dose to 200 J/cm^2^ (FI: 43.4 ± 13.9), indicating the dependence of ROS production on light dose.

### Visudyne®–PDT induces apoptosis of plaque cells

**Figure 6:**
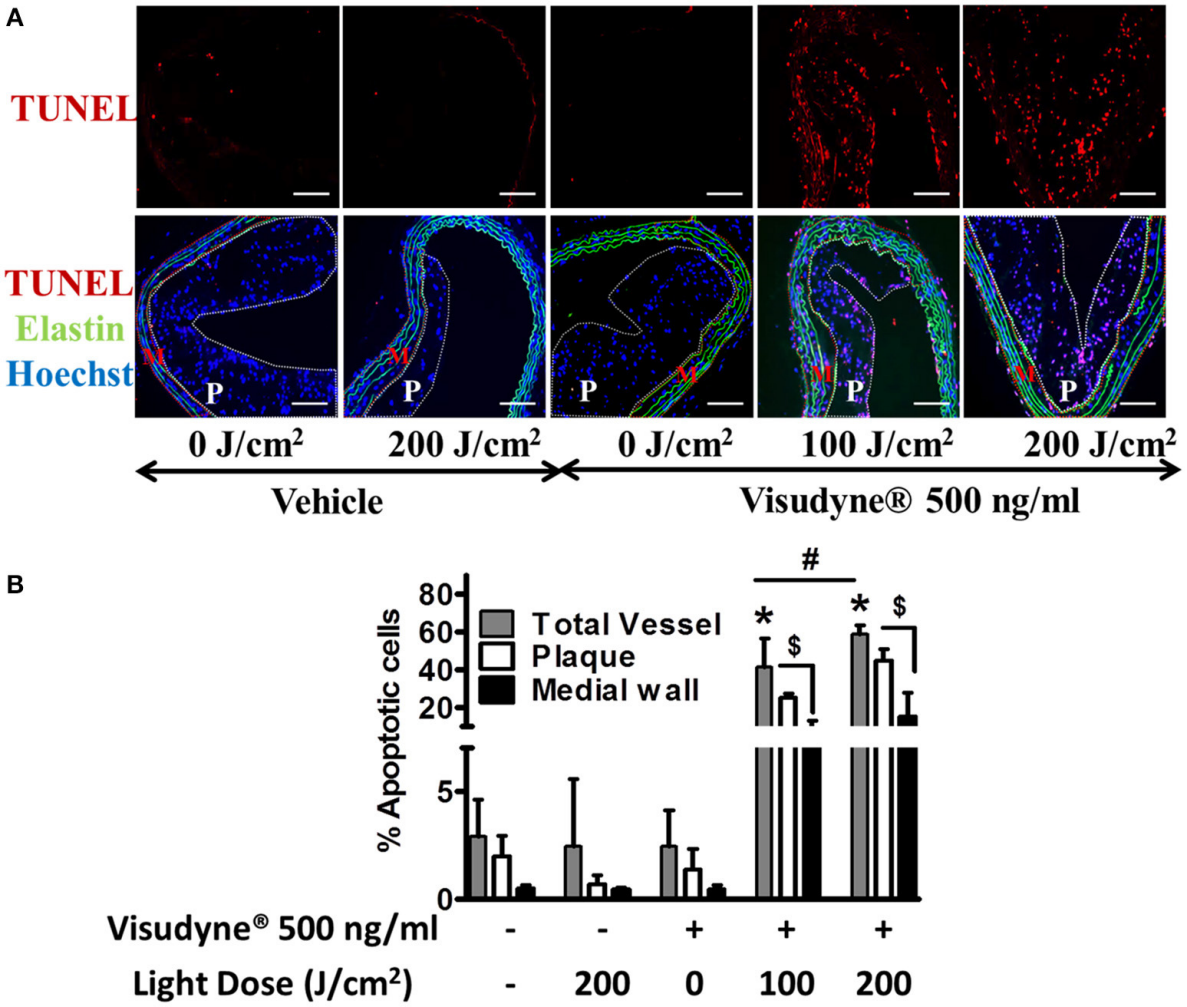
**Visudyne^®^-PDT induced apoptosis of plaque cells. (A)** TUNEL assay was performed after 24 h in arteries treated with vehicle (*n* = 5), laser only (*n* = 4), Visudyne® only (*n* = 6), Visudyne® treated, laser (100 J/cm^2^; *n* = 5), or Visudyne® treated, laser (200 J/cm^2^; *n* = 4) treated groups. Upper panel shows TUNEL positive cells and are indicated by red fluorescence and lower panel is merged images with Hoechst (blue) and elastin (green) auto fluorescence. The plaque area is encircled with white dotted line and marked as P, while the media beneath plaque is marked as M and encircled by red dotted line. Scale bar: 100 μm. **(B)** Bar diagram representing the percentage of TUNEL-positive cells in total vessel, plaque or in media beneath plaque. *P < 0.05 vs. vehicle treated groups, ^#^*p* < 0.01 100 vs. 200 J/cm^2^, ^$^*p* < 0.001 plaque vs. media beneath plaque.

After PDT, the aorta was incubated for 24 h to assess the influence of PDT on apoptosis (Figure 6A). Quantitative analysis of TUNEL staining demonstrated that the percentage of total apoptotic cells was not significantly different among vehicle treated laser irradiated groups (2.4 ± 3.1%) or only vehicle treated groups (2.9 ± 1.7%). A similar degree of apoptosis (2.5 ± 1.6%) was noted in Visudyne® treated groups when the irradiation dose was null indicating no dark cytotoxicity of Visudyne® (Figure 6B). A large increase in TUNEL positive cell percentage was noted in Visudyne® treated arteries illuminated at 100 J/cm^2^ (41.3 ± 15.3%). The percentage of apoptotic cells significantly increased while increasing the laser irradiation dose to 200 J/cm^2^ (58.9 ± 4.6%; Figure 6B). A more detailed examination revealed that apoptotic cell percentages were higher within the plaque, encircled and marked as P (1.3 ± 0.9, 25.2 ± 2.1, 44.9 ± 6.0%) as compared with medial wall beneath plaque, encircled and marked as M (0.5 ± 0.2, 8.5 ± 4.7, 15.3 ± 12.7%) at light doses of 0, 100 or 200 J/cm^2^, respectively (Figures 6A,B).

### Visudyne®-PDT preferentially affects plaque macrophages

**Figure 7:**
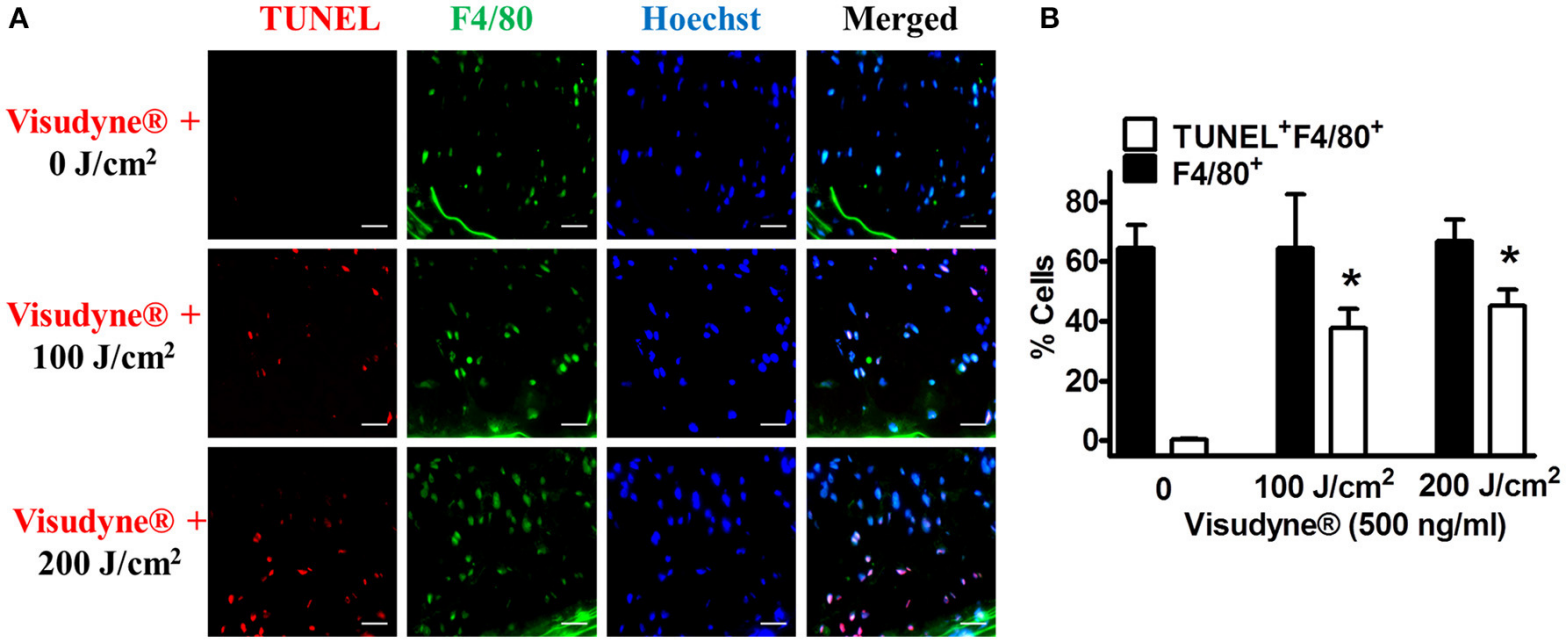
**Visudyne^®^-PDT mediated macrophage apoptosis. (A)** Representative images showing TUNEL-positive signal (green), F4/80-positive (red), Hoechst (blue), and merged image in Visudyne® treated arteries photoirradiated with 0 J/cm^2^ (upper panel, *n* = 3), 100 J/cm^2^ (middle panel, *n* = 3), or 200 J/cm^2^ (lower panel, *n* = 4). Scale bar: 25 μm. **(B)** Percentage of apoptotic macrophages was assessed as the percent of TUNEL^+^ F4/80^+^ cells to the total number of cells. *P < 0.001 vs. 0 J/cm^2^.

Immunostaining of macrophages using F4/80 antibody revealed high content of macrophages (63.4 ± 11.1%) within the plaques. Double immunofluorescence staining showed negligible apoptosis of macrophages in Visudyne® treated arteries that underwent no irradiation. Out of the total number of cells, only 0.3 ± 0.2% cells were F4/80^+^ TUNEL^+^ while most of the macrophages were not apoptotic i.e., F4/80^+^ TUNEL^−^ 64.3 ± 7.3% (Figures 7A,B). A significant increase in F4/80^+^ TUNEL^+^ cell population was evident in Visudyne® treated arteries illuminated with 100 J/cm^2^ (37.6 ± 6.4%) or 200 J/cm^2^ (45.3 ± 5.4%) leading to a significant reduction in non-apoptotic macrophages (26.9 ± 11.4%, 21.5 ± 2.3% at 100 J/cm^2^ and 200 J/cm^2^, respectively, Figures 7A,B). Importantly, macrophages contributed 80.1 ± 5.6% of total apoptotic cells in plaque, indicating that apoptosis of macrophages was more frequent after Visudyne®-PDT.

### Visudyne®-PDT attenuated vascular function

**Figure 8:**
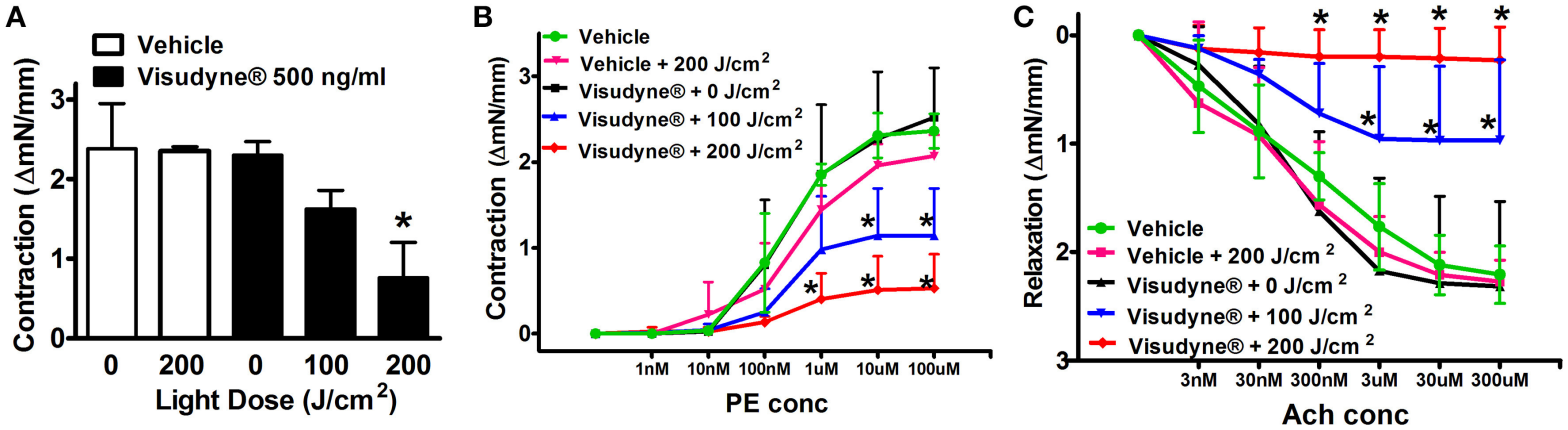
**Visudyne^®^-PDT reduces vascular function**. Aortic segments devoid of plaque from different treatment groups were mounted in Myograph. **(A)** Bar diagram representing KCl induced contraction **(B)** Dose-dependent phenylephrine (1 nM–100 μM) induced contractions and **(C)** Acetylcholine (3 nM–300 μM) mediated relaxations. Results are mean ± *SD* (*n* = 4). *p < 0.05 vs. Visudyne®.

The effect of PDT on vascular function was assessed 24 h after treatment using Myogram. Maximal tissue contractility induced by KCl was similar among vehicle only (2.4 ± 0.6 mN), laser only (2.4 ± 0.06 mN) and Visudyne® only (2.3 ± 0.2 mN) treated groups. A significant reduction in vasoconstriction was observed upon laser irradiation of Visudyne® treated arteries at 100 J/cm^2^ (1.6 ± 0.3 mN) or 200 J/cm^2^ (0.8 ± 0.4 mN; Figure 8A). Likewise PE induced maximal contraction was similar between vehicle only (2.4 ± 0.2 mN), Visudyne® only (2.5 ± 0.6 mN) and laser only (2.0 ± 0.3 mN) treated groups and were significantly reduced upon laser illumination of Visudyne® treated arteries at 100 J/cm^2^ (1.1 ± 0.5 mN) or 200 J/cm^2^ (0.5 ± 0.4 mN; Figure 8B). Similarly, Ach induced maximal relaxation was significantly reduced in Visudyne® treated arteries irradiated at 100 J/cm^2^ (1.0 ± 0.7 mN) or 200 J/cm^2^ (0.2 ± 0.3 mN) as compared to only Visudyne® treated groups (2.3 ± 0.8 mN). Ach induced vasorelaxation in laser alone (2.3 ± 0.2 mN) or vehicle treated groups (2.2 ± 0.3 mN) was comparable to only Visudyne® treated groups (Figure 8C). Percentage relaxation in Visudyne® treated arteries (87 ± 12%) was only slightly reduced upon laser irradiation at 100 J/cm^2^ (78 ± 19%) or 200 J/cm^2^ (72 ± 25%).

## Discussion

We report here that local arterial perfusion for a short period of time can lead to a high accumulation of Visudyne® in atherosclerotic plaque. Subsequent intra-arterial photo-activation of Visudyne® induces significant apoptosis of plaque macrophages. Moreover, Visudyne®-PDT attenuates the vascular functionality of smooth muscle cells. Therefore local Visudyne®-PDT could be effective for management of atherosclerosis and restenosis.

The role of PDT in the prevention of catastrophic events in atherosclerosis is well established. Previous studies utilizing Photofrin (Hsiang et al., 1995) or 5-aminolevulinic acid (Peng et al., 2011) have demonstrated that PDT can inhibit atherosclerotic plaque progression. An *in vivo* study conducted by Hayase et al. showed that Motexafin lutetium based PDT promotes stabilization by decreasing plaque macrophage content (Hayase et al., 2001). Photosensitizers are generally administered via systemic injection that may lead to inconvenient cutaneous photosensitivity such as erythema and oedema lasting up to 3 months, in some cases (Braathen, 2014), as well as limited accumulation in the specific region of interest. Selective accumulation of the photosensitizer is critically important for the success of PDT. As a result, the intra-arterial, local drug delivery approach has been chosen to minimize systemic exposure to the drug and to attain high therapeutic concentration of the photosensitizer directly on the plaque within short treatment time compatible with the surgical interventions.

Verteporfin is a potent, second-generation, FDA approved photosensitizer with minimal skin photosensitization. Verteporfin is activated by 690 nm photons, allowing for sufficient light penetration and drug activation. It binds with endogenous low density lipoproteins (LDL) and is significantly more cytotoxic against non-adherent cells as compared to Hematoporphyrin derivative (Richter et al., 1987). Previous reports suggest that Verteporfin-based PDT might prevent the development of intimal hyperplasia (Adili et al., 1998). However, studies evaluating anti-atherosclerotic efficacy were still lacking. In the present work, local delivery of Visudyne® into the plaque of atherosclerotic aorta has been achieved via an *ex vivo* approach based on a modified system described previously (Zulliger et al., 2002; Prasad et al., 2014). The *ex vivo* approach reduces the complexity of *in vivo* system and provides a near physiological environment. This *ex vivo* approach also enabled us to perform intra vascular light activation of the photosensitizer localized in the plaque. Since Visudyne® accumulation reaches saturation after only 15 min of treatment. We have therefore selected this relatively short time as optimal treatment time for the laser irradiation. DLI is an important parameter that governs the efficacy and selectivity of vascular damage that occurs during the treatment. A short DLI is clinically desirable to facilitate a single treatment in the patient or day-patient clinic that otherwise is limited to treating patients at some interval after angioplasty with possible undesired complications of repetitive invasive injury. Previously, P. Gonschior et al. demonstrated the possibility of reducing DLI using a local delivery approach (Gonschior et al., 1996). Local delivery of Photofrin (5 mg in 2 mL over 30 s) was performed in porcine arteries using a macro-porous balloon catheter. Intra-vascular illumination with an argon-dye laser reduces myointimal hyperplasia after arterial injury (Gonschior et al., 1996). Likewise Hayase M, et al. showed that activation of Motexafin lutetium after 15 min of local infusion reduces plaque burden. Motexafin lutetium was applied by using a drug delivery balloon catheter where it was infused for 15 min directly over the plaque in rabbit atherosclerotic artery (Hayase et al., 2001). Local delivery also enabled to cut the total dose required to about one tenth to that of systemic dose. The findings of the present study showed that local delivery can result in high accumulation of Visudyne® in the plaque within a short application time, thus supporting the feasibility of Visudyne® mediated PDT under clinical conditions.

Efficient and accurate delivery of light to the target tissues is particularly important. The best way to achieve suitable illumination is through endovascular light delivery. Endo-luminal photo activation of Visudyne® was achieved using a cylindrical light diffuser which being at the center of the vessel's lumen, thus providing a homogenous and precisely controlled light dosimetry. It should be noted that the distribution of the light throughout the thickness of the plaque is not fully uniform. This could also account for more apoptosis of plaque cells when the photosensitizer is activated via an inside-out approach.

Upon illumination, photosensitizer molecules absorb light and start reactions eventually generating hydroxyl radical, superoxide anion, hydrogen peroxide (Type I PDT), and singlet oxygen (Type II PDT). Singlet oxygen can further react with nearby molecules to induce the formation of other ROS species that could be detected via chemical sensors like DHE (Satoh et al., 2010; He et al., 2016). When ROS concentration surpasses a certain level it leads to apoptosis via loss of mitochondrial membrane potential, lipid peroxidation, or protein denaturation of cellular membranes and organelles (Chen et al., 2001; Wang and Yi, 2008). Increasing ROS levels is widely utilized in multiple therapeutic approaches like radiotherapy, chemotherapy and PDT for the destruction of leukemia cells (Chou et al., 2004; Wang and Yi, 2008) or macrophages (Zhu et al., 2015). The amount and micro-localization of ROS is dependent on the concentration and supply of oxygen in the tissue and possibly also on the irradiation intensity and fluence rate. This indicates that it is important to assess light dose effects for effective treatment. A light dose dependent increased ROS level leading to 50–60% apoptosis was noted following photo activation of Visudyne®. Our results demonstrate that locally delivered Visudyne® in the absence of light does not alter the apoptosis, indicating no dark toxicity of the photosensitizer. These findings are in agreement with previous reports where Verteporfin alone below 100 ng/ml concentration does not exerts any cytotoxic effects in Retinoblastoma Cell Lines (Stephan et al., 2008). Furthermore, our data show that Visudyne®-PDT resulted in a higher apoptosis of plaque cells as compared to part of the media which was covered by plaque. A possible explanation for such a differential effect could be made on the basis of inherent property of Verteporfin to preferentially localize in plaque (Hsiang et al., 1993; Allison et al., 1997). BA Allison et al. used a mutant fibroblast cell line which has no LDL receptors to demonstrate that LDL receptors are responsible for the accumulation of lipoprotein-associated Verteporfin (Allison et al., 1994). LDL receptors being present on macrophages (Hansson and Hermansson, 2011) may be involved in the uptake of Visudyne® which is a liposomal formulation of Verteporfin. PDT using porphyrins or texaphyrin derivatives like Lutetium texaphyrin, or 5-aminolevulinic acid, have previously been shown to reduce plaque macrophages resulting in stabilization of atherosclerotic plaques and reduction of plaque inflammation (Hayase et al., 2001; Peng et al., 2011). We therefore further investigated the effect of PDT on plaque macrophages. Our results demonstrated that the bulk of apoptotic cells were macrophages. Phagocytic cells like macrophages possess a powerful ROS generating system in response to stimulation with various agents (Forman and Torres, 2001; Swindle et al., 2002; Italiani and Boraschi, 2014). It could thus be assumed that macrophages were more efficient in PDT induced ROS production and were therefore more susceptible to apoptosis. Another possible explanation could in relation with the plaque composition. Atherosclerotic plaques in the present study were characterized by thin fibrous cap with high macrophage population (63% of total cells). Since PDT induces inflammation, which in turn causes the tight junctions between endothelial cells to let leukocytes pass, hence Visudyne® can easily get to the macrophages (with high probability to come in contact with the photosensitizer) in the plaque. Since the ROS produced by activated photosensitizer have very short diffusion distance (<100 nm), PDT induced apoptosis is restricted to cells with adequate presence of photosensitizer like the macrophages in this case.

During atherogenesis, SMC undergo phenotypic switching from the contractile to the synthetic state. SMC migrate from media to intima where they proliferate and contribute to other cellular processes such as inflammation, apoptosis, and matrix alterations (Dzau et al., 2002) that in turn aggravate atherosclerosis progression. Prominent vascular effects occur during and after PDT. Although there are no clear reports demonstrating the phenotypic modulation of SMC immediately after PDT, experimental evidence point to the fact that PDT by producing cytotoxic free radicals results in localized depletion of SMC (Granville et al., 2001) that eventually leads to initial reduction in plaque SMC content (Waksman et al., 2008; Peng et al., 2011). Such depletion of SMC in the medial wall is beneficial in preventing neointimal hyperplasia (Nyamekye et al., 1996; Nagae et al., 2001) or atherosclerosis (Peng et al., 2011). This is followed by repopulation of plaques with a non-proliferating quiescent SMC (Waksman et al., 2008). Moreover, PDT induced injury to SMC does not induce an inflammatory response in the vessel wall (Statius van Eps et al., 1997) but reduces basic fibroblast growth factor content thereby inhibiting invasive SMC mitogenesis to favorably alter the vascular injury response (Statius van Eps et al., 1997). We therefore examined the effect of Visudyne®-PDT on the viability and functionality of medial SMCs. Indeed, our data using Myogram demonstrated that the viability and contractility of SMCs was significantly reduced following Visudyne®-PDT. In the present study, we used Ach to study the influence of PDT on endothelium mediated relaxations. Ach receptors are expressed on endothelial cells of blood vessels and their activation results in increased intracellular calcium and generation of endothelium-derived relaxing factors (EDRF) such as nitric oxide which induce relaxation of the adjacent vascular SMC (Coats et al., 2001). Since phenylephrine induced smooth muscle contractions were substantially reduced following Visudyne-PDT, an expected reduction in Ach induced net relaxation was observed. However, it remains unclear whether such reduction in relaxation response is due to dysfunctional SMC or dysfunctional endothelium. We therefore calculated percentage relaxation by normalizing the net contraction induced by submaximal phenylephrine where a modest reduction in endothelium dependent vasorelaxation was observed. These results corroborated with previous reports where PDT using Photofrin as a photosensitizer impairs the production or release of EDRF by the endothelium (Gilissen et al., 1993). Previous reports also suggest that singlet molecular oxygen, superoxide anion radical, H_2_O_2_ and hydroxyl radical, generated during PDT are involved in not only supressing the noradrenaline induced contraction but also impairs endothelium-dependent relaxation evoked by acetylcholine (Mizukawa and Okabe, 1997). Our results indicate that Visudyne®-PDT could induce the apoptosis of plaque macrophages and at the same time could reduce the function of the vascular smooth muscle cells.

## Conclusion

This study demonstrated that local drug delivery can result in high accumulation of Visudyne® within atherosclerotic plaque shortly after the drug administration. Intra-arterial light activation induces significant apoptosis of plaque macrophages. A short DLI offered by local Visudyne® application can be effectively utilized for treatment of atherosclerotic plaque in conjunction with PCI procedures.

## Study limitations

The study was conducted in mouse model of atherosclerosis. Although the lesions of ApoE^−/−^ mice resemble many of the characteristic of human atheroma, yet the plaque stiffness, muscularity, and thickness vary to a great extent between mouse aorta and human coronary artery. Although previous reports documented the feasibility of intracoronary PDT in porcine model (Waksman et al., 2006) as well as in human subjects (Kereiakes et al., 2003), precautions must be taken to use drug delivery catheters and diffusers suitable to the geometry of the vessel particularly in the case of highly occluded vessels in order to allow smooth insertion and to avoid any mechanical damage to the vessel walls. Further studies in large animal models having plaque more similar to humans are therefore warranted to investigate long term effect in particular the late effects of Visudyne®-PDT in terms of plaque composition and plaque stabilization/regression.

## Author contributions

MJ, MZ, AF, JV, HB, GW, SC, and MG contributed to design of the work. MJ performed the experiments. AF planned and provided experimental animals. JV helped with microscopic acquisition of the images. MJ and MG analyzed the data and drafted the manuscript. MZ and GW provided light source and contributed to light dosimetry calculations. MZ, HB, GW, and MG provided critical inputs for revising manuscript. All authors approve the final version of manuscript for publication and agree to be accountable for all aspects of the work.

## Funding

This work was supported by the Swiss National Science Foundation Grant 150271.

### Conflict of interest statement

The authors declare that the research was conducted in the absence of any commercial or financial relationships that could be construed as a potential conflict of interest. The reviewer QY and handling Editor declared their shared affiliation, and the handling Editor states that the process nevertheless met the standards of a fair and objective review.
